# A Quantitative Method for Detecting Ara h 2 by Generation and Utilization of Monoclonal Antibodies

**DOI:** 10.1155/2018/4894705

**Published:** 2018-05-06

**Authors:** Huifang Chen, Zehong Zou, Ailin Tao

**Affiliations:** The Second Affiliated Hospital, The State Key Laboratory of Respiratory Disease, Guangdong Provincial Key Laboratory of Allergy & Clinical Immunology, Guangzhou Medical University, Guangzhou, China

## Abstract

Peanut (*Arachis hypogaea*) is one of the most common food allergens that can induce fatal anaphylaxis, and Ara h 2 is one of the major allergen components involved in peanut allergy. The aim of this study was to develop a quantitative method for detecting peanut allergen using monoclonal antibodies against Ara h 2. The splenocytes of immunized mice were fused with myeloma cells (SP2/0), and stable mAb-producing clones were obtained by limiting dilution. mAbs against Ara h 2 were isolated from mouse ascites, and specificity was confirmed by immunoblotting. Five mAbs with high purity and specific reactivity were obtained, which were referred to as 1-2E10, 2-1D5, 3-1C5, 4-1C2, and 5-1G4, respectively. After screening different mAb combinations for development of a sandwich ELISA, we selected 5-1G4 as the capture antibody and 1-2E10 as the detection antibody for the measurement of Ara h 2 from which an optimal correlation between the Ara h 2 concentration and the OD value was obtained. This sandwich ELISA could specifically detect Ara h 2 in peanut extract at concentrations as low as 5 ng/mL and up to 10 *μ*g/mL. These mAbs can, therefore, serve as quantitative diagnostic reagents for peanut and peanut product risk assessment.

## 1. Introduction

The incidence of food allergy has been increasing in developing countries in recent decades and now affects up to 10% of the world population [1]. Food allergy-associated diseases have become a global health problem and a major food safety concern [2]. The gastrointestinal symptoms of food allergy include stomach pain, nausea, vomiting, anorexia, abdominal distension, abdominal pain, diarrhea, mucus production, and so on. Peanut allergy has been recognized as one of the most severe food allergies that can lead to life-threatening reactions after unintentional consumption [3]. Currently, the only effective treatment for peanut allergy is to avoid exposure to peanut allergens. However, avoidance is very difficult since peanut allergens are commonly found in many food products. Many foods are inadequately labeled and do not inform the consumer that peanut products might be present and, therefore, can lead to the accidental ingestion of peanut allergens. Moreover, in places where peanuts are cooked and consumed, peanut allergens can diffuse into the environment. Peanut-allergic patients often feel anxious because of the fear of accident consumption or exposure that commonly occurs and could send them into a medical emergency. Some parents with peanut-allergic children demand their children to avoid all food nuts, to the extreme of restricting the consumption of foods outside the home resulting in a deprivation of many childhood pleasures [4].

Peanut allergy is one of the prime causes of anaphylactic deaths, especially in Western countries [5]. Approximately 3~5% children suffer from peanut allergy beginning in infancy and up to adulthood [6, 7]. Peanut allergens can elicit a more severe reaction than allergens from other legumes [8]. At least 18 potentially important peanut allergens have been identified and listed in the World Health Organization/International Union of Immunological Societies (WHO/IUIS) allergen database, termed as Ara h 1 to Ara h 17 plus peanut oleosin [9, 10]. Based on the criteria suggested by the WHO/IUIS Allergen Nomenclature subcommittee in 1994 [11], Ara h 1, Ara h 2, and Ara h 3 were identified as the major peanut allergens. Ara h 2 belongs to the conglutinate family of seed storage proteins and is related to the 2S albumin family [12, 13]. Compared with Ara h 1 and h 3, Ara h 2 has a much higher potency in degranulation assays, suggesting that Ara h 2 is the most potent allergen in causing sensitization in the peanut-allergic patient [9, 14].

There are two Ara h 2 isoforms, Ara h 2.01 and Ara h 2.02, with Ara h 2.02 being the more sensitive diagnostic reagent due to its additional IgE binding epitopes compared to Ara h 2.01 [15]. In this study, we generated mAbs against rAra h 2.02, which has the same sequence with that of seed storage protein SSP1 of *Arachis hypogaea* (GenBank accession number AAT00598.1). The mAbs were used to develop a sandwich ELISA for quantification of Ara h 2 in peanut extract. This sandwich ELISA system can be used to precisely measure the amount of Ara h 2 in immunotherapy reagents. It may be also be used for standardizing clinical reagents, for monitoring of the allergen level in foods and the environment that allergic patients are exposed to daily, and for creating and ensuring a low-level allergen environment for patients; all of which could be beneficial to these peanut-allergic patients.

## 2. Material and Methods

### 2.1. Materials

All procedures performed in this study involving animals were in accordance with the ethical standards of the institution and have been approved by the research ethics committee of the Second Affiliated Hospital of Guangzhou Medical University and conform to the Guiding Principles in Use and Care of Animals published by the National Institutes of Health [16]. Balb/C mice were purchased from the Medical Laboratory Animal Center (Guangdong, China). Complete Freund's adjuvant (CFA) and Incomplete Freund's adjuvant (IFA) were purchased from Sigma-Aldrich Co. (St. Louis, USA). Sp 2/0 myeloma cell line was obtained from CAS Shanghai Life Science Cell Resource Center (Shanghai, China). HAT medium, HT medium, penicillin, streptomycin, fetal bovine serum (FBS), and RPMI-1640 medium plus L-glutamine were purchased from Life Technologies Inc. (New York, USA). Horse anti-mouse IgG-HRP conjugate was purchased from Cell Signaling Technology (Shanghai, China). Polyethylene glycol 4000(PEG 4000) was bought from Sigma-Aldrich Co. Strep-tag II column (5 × 1 mL Lot: 10183115) and HiTrap protein G (5 × 1 mL Lot: 17-0404-01) were bought from GE Healthcare (Freiburg, Germany). ISO-2 kit was purchased from Sigma-Aldrich Co. and the EZ-Link™ plus activated peroxidase kit from Thermo Scientific Co.

### 2.2. Generation of mAbs against Recombinant Ara h 2

Recombinant Ara h 2 (rAra h 2, referenced to GenBank accession number AAT00598.1) was expressed in *E. coli* and purified using a Strep-tag II column from inclusion bodies. Four 6–8-week-old Balb/C mice were immunized with purified rAra h 2 according to the procedure described previously [17, 18]. In brief, mouse mAbs against rAra h 2 were produced by the fusion of the myeloma cells sp2/0 and the spleen cells from the mice immunized with the purified rAra h 2 for three times at 2-week intervals. The hybridoma cells producing antibodies against rAra h 2 were screened by indirect ELISA and subcloned by limiting dilution to obtain stable mAb-producing cell lines. A commercial ISO-2 kit was used to identify the isotypes of the mAbs according to the manufacturer's instruction.

### 2.3. Purification and Characterization of mAbs

The stable mAb-producing cells were inoculated into the abdominal cavity of mice. The ascetic fluid was collected, and antibodies were purified using protein G affinity chromatography according to the manufacturer's instruction. The titers of the purified mAbs against Ara h 2 were measured by indirect ELISA, and the specificity was confirmed by immunoblotting (Supplementary Material 2.3).

### 2.4. Conjugation of Horseradish Peroxidase (HRP) to the mAbs

The Thermo Scientific™ EZ-Link plus activated peroxidase, an amine-reactive HRP, generated higher conjugate yields (>95%) than a method based on glutaraldehyde chemistry. The purified mAbs were dialyzed in carbonate-bicarbonate buffer (pH 9.4), and conjugation was carried out according to the manufacturer's recommendation.

### 2.5. Whole Peanut Extract

Peanut extract was made according to the protocol described by Caudrado et al. [19]. In brief, peanut flour from raw or roasted peanuts was suspended in a solution containing 100 mm Tris-HCl, 100 mm EDTA-Na_2_, and 100 mm NaCl at 1/10 *w*/*v*, rotated for 5 h at 4°C, and then centrifuged at 10,000*g*, 4°C for 20 min. The supernatant was collected and dialyzed in distilled water for 48 h at 4°C. The concentration of the protein was determined by BCA assay using bovine serum albumin (BSA) as the standard following the manufacturer's instruction.

### 2.6. Development of an ELISA for Ara h 2 Quantification

To build a sandwich ELISA, purified mAbs were used as the capture antibody to coat a polyvinyl microtiter plate (Costar, USA) with 100 *μ*L of 1 *μ*g/mL mAb solution. Purified rAra h 2 was used as the standard antigen, which was serially diluted from 10,000 ng/mL to 4.9 ng/mL, and the HRP-conjugated antibodies were used as the detection antibody. The optimal matching antibody pair was selected by comparing the correlation between the rAra h 2 concentration and the OD value of the different combinations of the 5 different antibodies.

### 2.7. Detection of the Cross-Reactivity of the mAbs

Since peanut allergens share significant amino acid similarity with antigens from other legumes and tree nuts, we further tested the specificity of the mAbs by assessing their cross-reactivity with other common food allergens by Western blotting. For this, we tested Ara h 8, Bet v 1, Gly m 4, and soybean extract as potential cross-reacting allergens with the Ara h 2 mAbs and used bovine serum albumin (BSA) as the negative control and peanut extract as the positive control. The HRP-conjugated mAbs were used as detection antibodies.

## 3. Results

### 3.1. Generation of mAbs against rAra h 2

**Figure 1:**
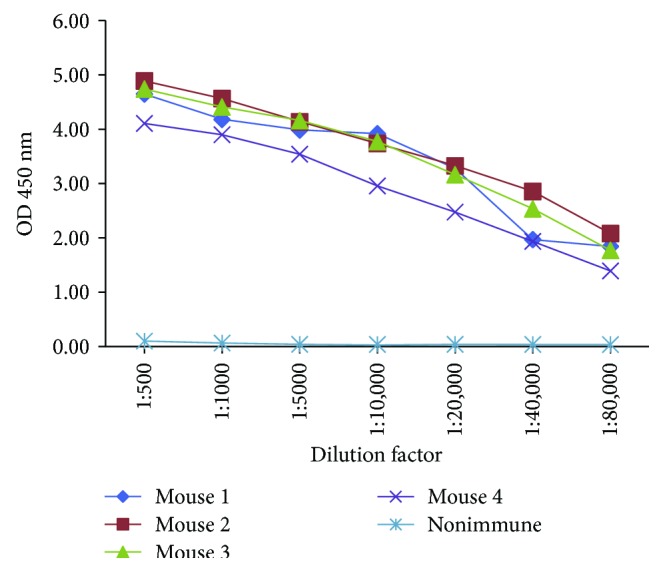
The serum titers of immunized mice.

After immunization, the sera from immunized and unimmunized mice were diluted at 1 : 500, 1 : 1000, 1 : 5000, 1 : 10,000, 1 : 20,000, 1 : 40,000, and 1 : 80,000 and the titers of rAra h 2-specific IgGs were measured by indirect ELISA. The results show that there were high titers of rAra h 2- specific antibodies in the sera of immunized mice (Figure 1). After fusion of splenocytes with the hybridoma cells, we obtained eight positive clones. After the first cycle of subcloning, five clones stably produced specific antibodies, while the other three clones were determined to be negative (Table 1). Four clones were IgG1 isotype while one clone was IgG2b isotype (Table 2).

**Figure 2:**
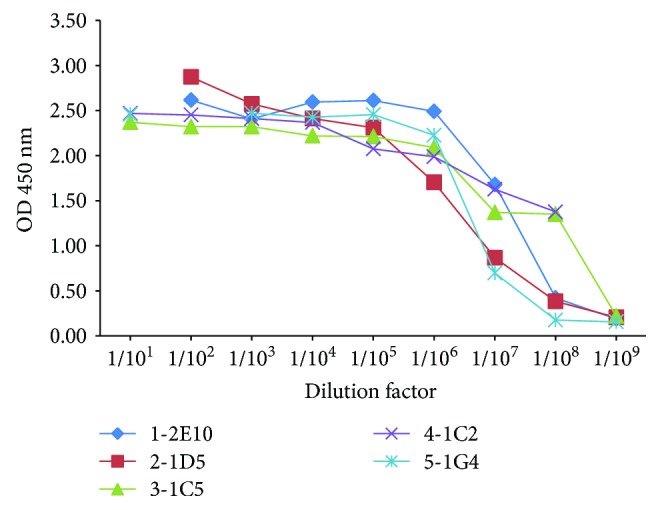
The mAb titers before purification (ascetic fluid).

The five mAb-producing clones were expanded and inoculated in Balb/C mice. The ascetic fluid was collected, and titration was performed by ELISA. The titers of the ascites were higher than 1 : 10^8^ (Figure 2) and, thus, adequate for use in the subsequent experiments.

### 3.2. Purification of the Ara h 2 mAbs

**Figure 3:**
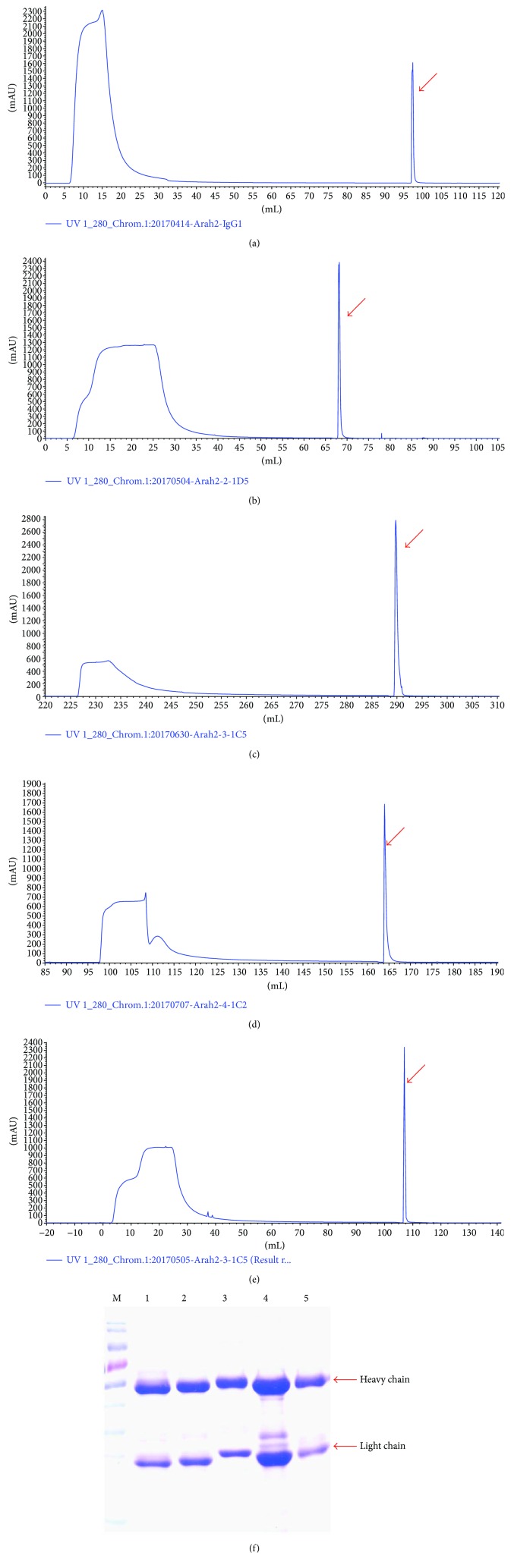
Purification of the mAbs against rAra h 2. (a) Clone 1-2E10 purification graph. (b) Clone 2-1D5 purification graph. (c) Clone 3-1C5 purification graph. (d) Clone 4-1C2 purification graph. (e) Clone 5-1G4 purification graph. (The arrows indicate the eluted peak of the target protein.) (f) The mAbs after purification. M: prestained protein marker. Lane 1: 1-2E10. Lane 2: 2-1D5. Lane 3: 3-1C5. Lane 4: 4-1C2. Lane 5: 5-1G4.

The Abs in the ascites were precipitated with 50% ammonium sulfate and purified by protein G affinity chromatography. As shown in Figures 3(a)–3(e), the first peak in the UV 280 graphs denoted the unbound protein and the second peak is the target protein (indicated with an arrow). The eluted proteins were subjected to SDS-PAGE. As shown in Figure 3(f), sharp individual bands of heavy chain and light chain antibody indicated that they are single clones.

### 3.3. Characterization of the Ara h 2 mAbs

**Figure 4:**
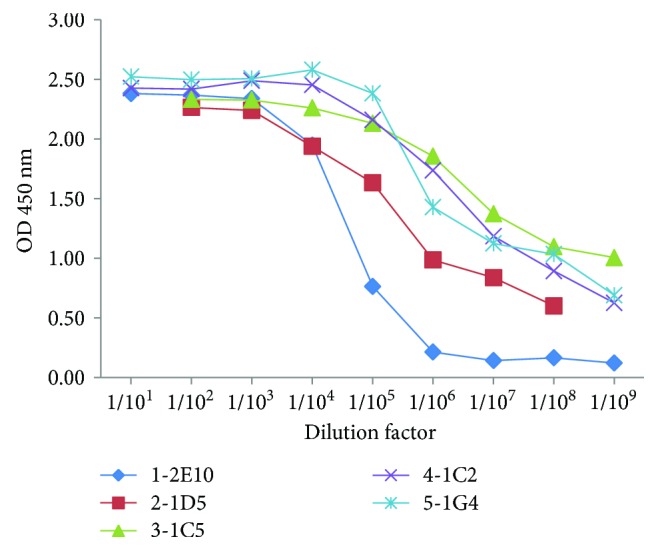
The mAb titers after purification.

**Figure 5:**
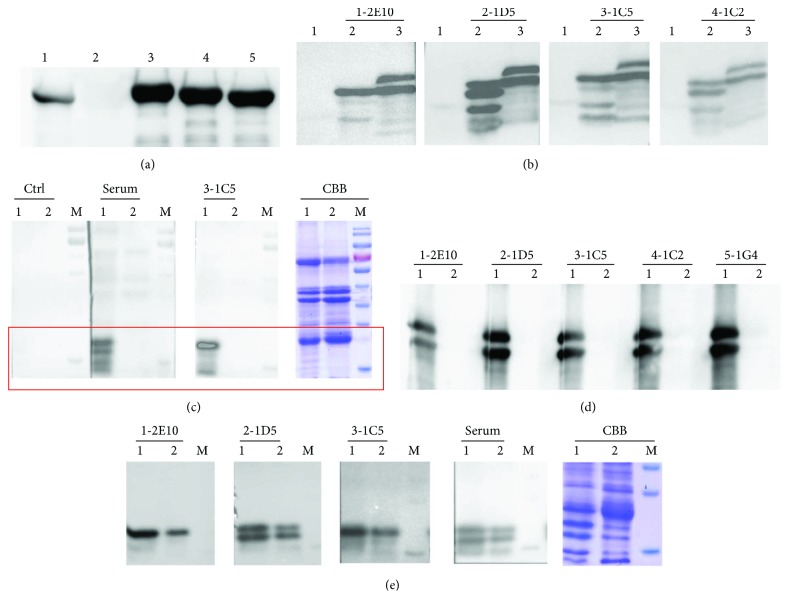
Characterization of the specificity of mAbs by Western blotting. (a) Isotyping of the IgG1 heavy chain of mAbs. Lane 1: 1-2E10-IgG1. Lane 2: 2-1D5-IgG2a. Lane 3: 3-1C5-IgG1. Lane 4: 4-1C2-IgG1. Lane 5: 5-1G4-IgG1. (b) Characterization of the specificity of the Ara h 2.02 mAbs. Lane 1: protein marker; lane 2: peanut extract before desalting; lane 3: rAra h 2; 1-2E10, 2-1D5, 3-1C5, and 4-1C2 were the mAbs applied individually. (c) Lane 1: peanut extract before desalting; lane 2: peanut extract after desalting; M: prestained protein marker; Ctrl: nonimmune serum; Serum: immunized serum; 3-1C5: the mAb was applied; CBB: Coomassie brilliant blue staining. (d) Lane 1: rAra h 2; lane 2: peanut extract after desalting; 1-2E10, 2-1D5, 3-1C5, 4-1C2, and 5-1G4 were the mAbs applied individually. (e) Lane 1: roasted peanut extract; lane 2: raw peanut extract; M: prestained protein marker; 1-2E10, 2-1D5, 3-1C5, and serum (immune serum) were applied individually. CBB: Coomassie brilliant blue staining.

After purification, the mAb titers were measured by indirect ELISA. As shown in Figure 4, the titers were higher than 1 : 10^8^, indicating highly efficient purification of mAbs. To confirm the isotypes and the specific reactivity of the mAbs against Ara h 2, Western blotting was performed. The isotype of the heavy chain was verified by goat anti-mouse IgG1-heavy chain (HRP) antibody. As shown in Figure 5(a), four mAbs were of the IgG1 isotype and only the 2-1D5 mAbs was of the IgG2a isotype. All mAbs recognized both recombinant Ara h 2 and native Ara h 2 in the peanut extract (Figure 5(b)). However, neither the mAbs nor the immune sera were able to recognize the peanut extract protein after desalting (Figures 5(c) and 5(d)), indicating that the desalting procedure may have affected the configuration and antigenic epitopes of the allergen. We then compared the mAb reactivity toward Ara h 2 in roasted and raw peanut extract. The result showed that the mAbs can recognize the allergen from both sources (Figure 5(e)).

### 3.4. Quantification of Ara h 2 by a Sandwich ELISA System

**Figure 6:**
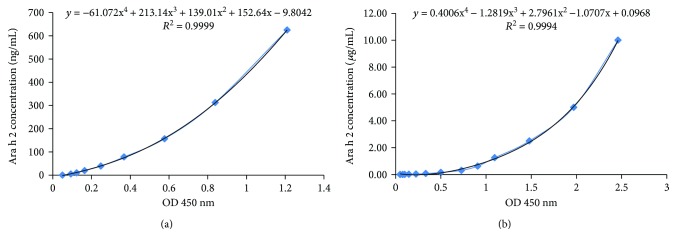
The correlation between the rAra h 2 concentration and the OD value at 450 nm (*R* ^2^ = 0.9994). (a) Standard curve between 5 ng/mL and 625 ng/mL. (b) Standard curve between 5 ng/mL and 10 *μ*g/mL.

To develop a sandwich ELISA system, a purified mAb was used as the capture antibody and a different conjugated mAb was used as the detection antibody. After screening, 5-1G4 (1 *μ*g/mL) was selected as the capture antibody and the HRP-conjugated 1-2E10 (at 1 : 2000 dilution) was chosen as the detection antibody. The correlation between the concentration of rAra h 2 and the OD value at 450 nm showed an excellent correlation (*R* ^2^ = 0.9999) (Figure 6(a)). The concentration range of Ara h 2 detectable in peanut extract was from 5 ng/mL to 625 ng/mL. Moreover, the correlation was still very high, up to 10 *μ*g/mL (*R* ^2^ = 0.9994) (Figure 6(b), Supplementary Material Table 1), demonstrating the superb sensitivity and detection range of this ELISA system.

### 3.5. Cross-Reactivity of the mAbs

**Figure 7:**
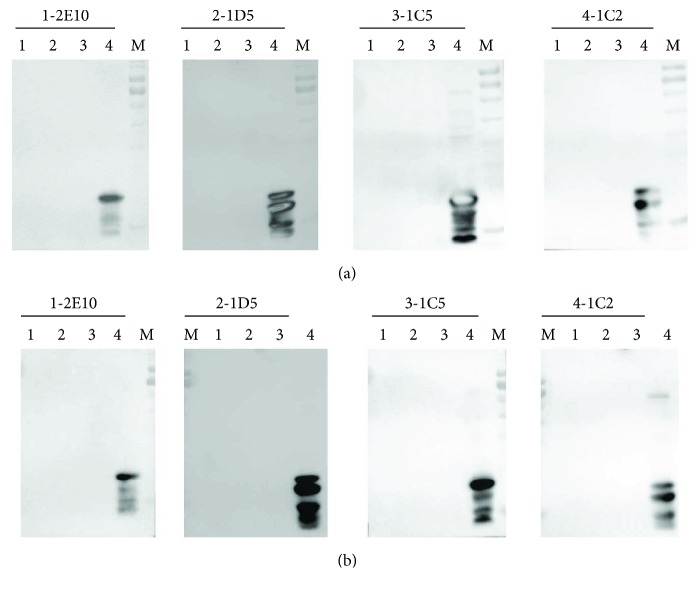
Evaluation of the cross-reactivity of the mAbs by Western blotting. (a) Lane 1: Ara h 8; lane 2: Bet v 1; lane 3: negative control (BSA); lane 4: positive control (peanut extract); M: prestained protein marker. (b) Lane 1: Gly m 4; lane 2: soybean extract; lane 3: negative control (BSA); lane 4: positive control (peanut extract); M: prestained protein marker.

We selected Ara h 8, Bet v 1, Gly m 4, and soybean extract to examine potential antibody cross-reactivity, using BSA as the negative control and peanut extract (not desalted) as the positive control. The mAbs did not cross-react with any antigens except for Ara h 2 (Figure 7). Therefore, the mAbs can specifically distinguish Ara h 2 from the other food allergens tested.

## 4. Discussion

Quantification of the allergen level in foods and the environment is useful for preventing allergen exposure and standardizing immunotherapy reagents [20]. Here, we developed a sandwich ELISA to quantify the peanut allergen Ara h 2 by using mAbs raised against recombinant Ara h 2. All the mAbs created showed good reactivity to both rAra h 2 and peanut extract from both roasted and raw peanuts. However, they did not recognize peanut extract after the desalting process. This suggested that the desalting procedure might have changed the protein conformation, most likely the antigenic epitope-containing domains, or that Ara h 2 may have been lost during the desalting step due to its low molecular weight [21]. In any case, the mAbs recognized Ara h 2 from both raw and roasted peanuts, indicating that the antigen epitopes are quite stable.

Peanut-allergic patients can experience symptoms from urticaria to anaphylaxis and a significant decrease in their quality of life. Allergen immunotherapy (AIT) is an effective treatment for allergies since it can modify the immune system by shifting a Th2 response to a Th1 response, inducing anergy in epitope-specific T cells, and regulating differentiation of allergen-specific T cells [22–24]. Currently, whole peanut extract is the most commonly used reagent for skin prick test (SPT) and AIT study, which can potentially elicit adverse events including anaphylaxis [25]. Furthermore, the strategies of peanut immunotherapy have varied from subcutaneous, epicutaneous, and sublingual application to oral treatment but none of them are recommended for routine clinical usage because of the different dosing schedules and the varying duration of treatment [26, 27]. Here, we established a new method to quantify Ara h 2 using purified mAbs against the allergen, which may help to standardize other immunotherapeutic reagents. In addition, we propose a new treatment strategy that uses purified mAbs against rAra h 2 to block the systemic dispersion of the allergen, which may reduce or prevent the hypersensitivity reaction in subjects.

There are at least 18 allergen components in peanut; Ara h 1, Ara h 2, and Ara h 3 are the major allergens and Ara h 8 is defined as the minor allergen, and these share a significant amino acid similarity with the allergens Gly m 4 and Bet v 1 [28, 29]. Bet v 1 is the major allergen component of birch pollen, and a number of reports have been published on its cross-reactivity with peanut allergens [30, 31]. Peanuts and soybeans are frequently mixed consciously and unconsciously in foods made at home and in restaurants. Therefore, it is important and meaningful to specifically quantify the amount of potent allergens in foods apart from their cross-reacting allergens. The specificity of the mAbs against Ara h 2 is high enough to distinguish Ara h2 from the other common food allergens in mixtures. This could help the patient from ingesting peanut allergen, while reducing unnecessary avoidance.

The prevalence of peanut allergy has been steadily increasing over the past decade [32]. This is partially due to the peanut allergens hidden in various processed foods or prepared dishes. A study showed that the amount of peanut allergen in household dust is associated with peanut sensitization or peanut allergy in patients with atopic dermatitis, especially when exposure to the environmental allergen occurs within the first year of life through skin lesions or inhalation of dust particles [33]. Many sources of peanut allergens exist in our environment, such as peanut butter, household dust, and steam from food preparation in the kitchen, dining table surfaces, and even the sofa [34]. Data show that 50 to 100 peanut-allergic patients in the world will die annually because of accidental ingestion of peanut-contaminated food [35]. Therefore, for peanut allergy sufferers, it is important to know the presence of peanut allergen in foods and in the environment. Here, we have established a method to quantify the major peanut allergen Ara h 2, which would be helpful for peanut-allergic patients to avoid allergen exposure, as well as help improve their quality of life.

Food allergy is an IgE-mediated immune response disorder that involves many immune cells during the course of the disease [36, 37]. When constantly exposed to food allergens, chronic inflammation can develop in the intestinal tract. Mast cells, the main reactive immune cell in allergies, are also commonly found in tumor tissues [38]. Some preclinical studies have suggested that mast cells may contribute to tumor progression and that the inflammatory microenvironment could affect suppression of antitumor immunity [39]. We have detected and quantified the amount of Ara h 2 in intestinal samples by utilizing the mAbs produced in this study, and the preliminary results showed that there is a difference in Ara h 2 levels between precancerous and cancerous tissues (data not shown), thereby inviting further study.

## 5. Conclusion

We generated and purified five mAbs with highly specific reactivity toward Ara h 2 and established a quantitative ELISA by utilizing these mAbs to detect the level of Ara h 2 in the whole extract of both raw and roasted peanuts. This quantitative system can be utilized for standardization of allergen reagents, as well as for adequate food labeling and environmental allergen monitoring, which will help peanut-allergic patients avoid this dangerous allergen and increase the quality of their lives.

## Figures and Tables

**Table 1:** Positive clones selected after fusion and the first subcloning.

Clones selection after fusion		Clones selection after the first subcloning	
Clones	OD at 450 nm	Clones	OD at 450 nm
Neg. ctrl	0.051	Neg. ctrl	0.050
Pos. ctrl	3.46	Pos. ctrl	2.360
1-E4	3.042	1-1E4	2.26
4-B10	3.051	4-1B10	1.796
5-E2	3.867	5-1E2	2.233
5-E12	3.654	5-1E12	2.133
6-G9	3.676	6-1G9	2.4
8-D10	3.642	8-1D10	0.057
8-F8	3.846	8-1F8	0.055
8-H11	3.533	8-1H11	0.07

Neg. ctrl: negative control, serum from nonimmunized mouse. Pos. ctrl: positive control, serum from immunized mouse.

**Table 2:** Ara h 2 mAb isotyping ELISA at OD 450 nm.

Clone number	IgG1	IgG2a	IgG2b	IgG3	IgM	IgA
1-2E10	2.447	0.335	0.337	0.344	0.358	0.353
2-1D5	0.246	1.279	0.243	0.242	0.236	0.242
3-1C5	2.3	0.414	0.404	0.377	0.364	0.369
4-1C2	1.947	0.342	0.343	0.343	0.34	0.357
5-1G4	1.951	0.379	0.381	0.36	0.336	0.375
Medium	0.109	0.101	0.113	0.122	0.133	0.116

## Supplementary Materials

Supplementary Materials Table 1: the correlation between the concentration of rAra h 2 and the OD value at 450 nm.
