# Supplementary material for: A Quantitative Method for Detecting Ara h 2 by Generation and Utilization of Monoclonal Antibodies
Source: J Immunol Res. 2018 May 6;2018:4894705. doi: 10.1155/2018/4894705 (PMC5960532; doi:10.1155/2018/4894705)
Supplement: Supplementary Materials — Table 1: the correlation between the concentration of rAra h 2 and the OD value at 450 nm. [file 4894705.f1.pdf]

A quantitative method for detecting Ara h 2 by generation and utilization of monoclonal antibodies--supplementary material

2. Method

2.1 Specific antibody titration of immunized serum

ELISA plates were coated with purified rAra h 2 protein (20 ng/ml) and incubated at 4°C overnight. After washing four times with PBS containing 0.05% Tween 20 (PBS/T), the plates were blocked with 2% BSA/PBS and washed with PBS/T. 100 µl of diluted serum was added to each well and incubated for 1 h at 37°C. After washing, the secondary antibody was added (horse anti-mouse IgG at 1:4000 dilution) to each well and incubated for 1 h at 37°C. After washing, the assay was developed with TMB and the absorbance was read using an ELISA Reader at 450 nm.

2.2 Screening of antibody-producing cells

The ELISA procedure was the same as above except that the hybridoma supernatant was used instead of serum. BSA was used as the negative control and the immunized serum diluted at 1:10,000 as the positive control.

2.3 Characterization of the mAb by Immunoblotting

Ara h 2 /peanut extract protein was subject to SDS-PAGE and then transferred onto a PVDF membrane. The membrane was then blocked in 2% BSA for 2 h at room temperature. The Ara h 2 antibodies were diluted and added as the primary antibody. After incubation and washing step, goat anti-mouse IgG antibody (1:4000) was added as the secondary antibody. After the incubation and washing, the membrane was developed in ECL solution and the images were acquired using Image Quant LAS 4000 mini.

2.4 Running SDS-PAGE gel

The prepared gel was placed into the gel rig and running buffer added into the upper and lower chamber. The samples were diluted in loading buffer and heated in boiling water for 5 min, then loaded in the gel and run at 80 v for 20min, followed by running at 150 v for 50 min until bromophenol blue reached the bottom of the gel.

3.1 Result

Table 1: The correlation between the concentration of rAra h 2 and OD at 450 nm

rAra h 2 (μg/mL)	OD1	OD2	OD AVE.	SD
0.0000	0.050	0.050	0.050	0.000
0.0049	0.078	0.093	0.086	0.011
0.0098	0.101	0.123	0.112	0.016
0.0195	0.147	0.164	0.156	0.012
0.0391	0.225	0.248	0.237	0.016
0.0781	0.334	0.368	0.351	0.024
0.1563	0.500	0.577	0.539	0.054
0.3125	0.728	0.839	0.784	0.078
0.6250	0.908	1.210	1.059	0.214
1.2500	1.096	1.327	1.212	0.163
2.5000	1.479	1.627	1.553	0.105
5.0000	1.970	2.071	2.021	0.071
10.0000	2.461	2.564	2.513	0.073
				R^2=0.9994
